# Hospital discharge planning and continuity of care for aged people in an Italian local health unit: does the care-home model reduce hospital readmission and mortality rates?

**DOI:** 10.1186/1472-6963-9-22

**Published:** 2009-02-04

**Authors:** Gianfranco Damiani, Bruno Federico, Antonella Venditti, Lorella Sicuro, Silvia Rinaldi, Franco Cirio, Cristiana Pregno, Walter Ricciardi

**Affiliations:** 1 Department of Public Health, Catholic University of Sacred Heart, Largo F.Vito, 1, 00168, Rome, Lazio, Italy; 2 Faculty of Health and Sport Sciences, University of Cassino, Cassino, Lazio, Italy; 3 Territorial geriatrics unit, Local Health Unit n°4 of Turin, Turin, Piedmont, Italy; 4 Social services unit, Turin, Piedmont, Italy

## Abstract

**Background:**

Hospital discharge planning is aimed to decrease length of stay in hospitals as well as to ensure continuity of health care after being discharged. Hospitalized patients in Turin, Italy, who are in need of medical, social and rehabilitative care are proposed as candidates to either discharge planning relying on a care-home model (DPCH) for a period of about 30 days, or routine discharge care. The aim of this study was to evaluate whether a hospital DPCH that was compared with routine care, improved patients' outcomes in terms of reduced hospital readmission and mortality rates in patients aged 64 years and older.

**Methods:**

In a retrospective observational cohort study a sample of 380 subjects aged 64 years and over was examined. Participants were discharged from the hospital S.Giovanni Bosco in Turin, Italy from March 1^st^, 2005 to February 28^th^, 2006. Of these subjects, 107 received routine discharge care while 273 patients were referred to care-home (among them, 99 received a long-term care intervention (LTCI) afterwards while 174 did not). Data was gathered from various administrative and electronic databases. Cox regression models were used to evaluate factors associated with mortality and hospital readmission.

**Results:**

When socio-demographic factors, underlying disease and disability were taken into account, DPCH decreased mortality rates only if it was followed by a LTCI: compared to routine care, the Hazard Ratio (HR) of death was 0.36 (95% Confidence Interval (CI): 0.20 – 0.66) and 1.15 (95%CI: 0.77 – 1.74) for DPCH followed by LTCI and DPCH not followed by LTCI, respectively. On the other hand, readmission rates did not significantly differ among DPCH and routine care, irrespective of the implementation of a LTCI: HRs of hospital readmission were 1.01 (95%CI: 0.48 – 2.24) and 1.18 (95%CI: 0.71 – 1.96), respectively.

**Conclusion:**

The use of DPCH after hospital discharge reduced mortality rates, but only when it was followed by a long-term health care plan, thus ensuring continuity of care for elderly participants.

## Background

Intermediate care is aimed to facilitate transition from hospital to home when the objectives of care are not primarily medical: patients are discharged earlier, and hospital length of stay is decreased [1]. In line with the principle of 'care closer to home', intermediate care services should generally be provided in community-based settings, in the patient's home, or they may be provided in discrete step-down facilities on acute hospital sites [2].

The need for effective discharge planning for elderly patients is becoming increasingly important due to the rising number of elderly people requiring hospital care, pressure on beds and recognition of the problems surrounding hospital discharge [3]. The main problems concern poor communication between hospital and community [4-8], lack of assessment and planning for discharge [4,9] and inadequate notice of discharge to the patients [9,10]. Furthermore, discussion of discharge with patients and their caregivers has been generally infrequent [10,11]. Over-reliance on informal support and/or poor statutory service provision [10,12-14], lack of attention to the individual needs of the most vulnerable [9,15], and wasted or duplicated visits by community nurses [5] were reported in the literature.

Discharge planning aims to review current medication, facilitate compliance with established treatment, improve home functioning and safety, prevent unnecessary hospital admission, and promote effective rehabilitation services. It also aims to enable early discharge from hospital and prevent premature or unnecessary admission to long-term residential care. In Italy, this form of discharge is put into effect in Local Health Units, public enterprises which are legally creatures of the regions with administrative and financial independence [16,17]. In the Piedmont region, where the local population is 4,250,775 and the number of elderly is about 900,000 (21.2%), the problem of continuity of care for aged patients is particularly relevant [18]. In each Local Health Unit of Piedmont, an Operative Care Centre aims at managing community hospital, residential and home care services after hospital discharge of these patients [19]. Patient problems during post-discharge may vary over time and are often accompanied by unmet needs. These problems may be related to their physical, functional, emotional and social status and include patient-related factors, care-related factors and features related to the social network of the patient. The patient-related problems are associated with a decline in physical health status (such as physical complaints), with decreased functional status (difficulty in performing the activities of daily life, and/or need of care with these activities), or with disturbed emotional status (feeling insufficiently informed or having uncertain, negative feelings and emotional worries). Examples of health care related factors are the way in which patients and their family are prepared for discharge and the post-discharge period, home care is provided, and the extent to which hospital and home-care are inter-related. Finally, features related to the social network are the availability, the skills and the willingness of the social network to provide support and/or help for the patients. In all these areas patients might experience insufficient or inadequate support in coping with the difficulties or limitations involved, which can sometimes result in hospital readmissions. Problems after discharge and the influencing factors are interrelated, in concept and over time. Furthermore, the literature shows that post-discharge problems can be reduced by efficient discharge planning during hospitalization and by intensive after care, and that the risk of post-discharge problems can be predicted to some extent [20].

So far, most research has focused on the effectiveness of hospital discharge planning based on interventions delivered at home that are compared to routine care. Among patients with hip fractures discharged from a medical centre in northern Taiwan, it was found that patients who received hospital discharge planning had a shorter length of stay, lower rate of readmission, and higher survival rate compared to those who received routine care [21]. Phillips *et al*.'s meta-analysis indicated that patients undergoing post-hospital discharge planning had lower mortality or readmission rate (for the combined end-point, Relative Risk (RR) = 0.73; 95% CI 0.62–0.87) [22] compared to those receiving routine care. In the Parker *et al*. review, readmission's RR was 0.85 (95% CI, 0.76–0.95), indicating a reduction in relative risk of being readmitted for patients receiving post-hospital discharge protocols [23].

On the basis of fifteen reviews, Mistiaen *et al *concluded that there is only limited evidence for the positive impact on readmission rates of discharge interventions. Discharge interventions did not appear to be effective for three reviews in which the largest effects were observed when interventions from the discharge planning and discharge support side were combined across the hospital-home interface. In addition, two reviews showed that educational interventions might have some effect on aspects of the emotional status after discharge, on knowledge and medication adherence [20]. The limited evidence about effectiveness of discharge interventions may be due to the heterogeneity of studies. In addition, Shepperd *et al*. found that there were no statistically significant differences in mortality (Odds Ratio (OR) = 1.44, 95% CI 0.82–2.51) and readmission between discharge planning and routine discharge care (OR = 0.91, 95% CI 0.67–1.23) [24]. There was, however, some evidence that services combining needs assessment, discharge planning and a method for facilitating the implementation of these plans were more effective than services that do not include the latter action [25].

These findings indicate that discharge planning is likely to play a key role as a management tool in intermediate care when the latter is provided at patient's home. The National Health Service in the UK has recently commissioned an evaluation of intermediate care for older people: current evidence suggested substantial changes in service organization and provision, and favourable experience reported by the users [26].

However, since there is a wide diversity of provision of services and the lack of a standard terminology for what constitutes recovery or rehabilitation, there have been relatively few studies that specifically focus on the effectiveness of intermediate care in residential settings. As pointed out by Plochg et *al*., the setting up of intermediate care may encounter several difficulties [27]. No significant differences in mortality or hospital re-admission were found for subjects who were treated in an intermediate residential setting for 6 weeks compared to those who received routine care at home [28]. Consequently, the aim of this study was to evaluate whether a hospital discharge planning in a care-home setting (DPCH), compared with routine care, improved patient's outcomes. More specifically, our aim was to evaluate the effectiveness of DPCH in terms of reduced hospital readmission and mortality rates in patients aged 64 years and over in one of the main Local Health Unit of Piedmont, Italy.

## Methods

### Study Design

In this retrospective observational cohort study, we focused on a sample of 380 subjects aged 64 years and older who were discharged from the hospital "S. Giovanni Bosco" in Turin, Italy. Among these patients, 273 received a hospital discharge planning in a DPCH, while 107 patients received routine discharge care. We included in the study patients discharged between March 1^st^, 2005 and February 28^th^, 2006, whose age at hospital discharge was 64 years and over. All patients were discharged alive from the hospital and they were observed for a minimum of six months.

Before hospital discharge, a team composed of a geriatrician, a district nurse and social workers determined the medical, psychological, and functional capabilities of the elderly person in order to develop an integrated plan for treatment and follow-up after hospital discharge [29-32]. The evaluation was carried out through the use of instrumental scales (Activity of Daily Living – ADL, Instrumental ADL – IADL, Short Portable Mental Status Questionnaire – SPMSQ) and it was needed to identify the level of complexity of care. In addition, an evaluation of the presence of care givers, family network, presence of voluntary association and housing conditions lead to the definition of the appropriate social care. After the need of medical, social and rehabilitative treatments were taken into account, patients were assigned to receive either DPCH for about 30 days [19] or routine care at home. Patients were referred to DPCH mainly when there was the need of monitoring the effect of new prescribed therapies and/or they needed physical rehabilitation. The care-home setting consisted of 2 residential homes, with a total of 43 beds.

For subjects who received DPCH, individualised care pathways were provided by a multidisciplinary team involving nurses, physical therapists, occupational therapists, geriatricians, community care officers and social workers on a 24-hour basis. A nurse "case manager" was in charge of patient safety and monitored the implementation of the care plan, with the aims of improving patients' level of autonomy and supporting the creation of an adequate care network. Physical and occupational therapists were mostly engaged in developing patients' skills for daily living activities. After a period of about 30 days, a further Operative Care Centre assessment was performed. The pre-post comparison of scores for DPCH patients showed a slight improvement, especially for IADL scores, although this was not statistically significant at the 0.05 level. Patients could then be entitled to receive a long-term care intervention (LTCI) within the same residential setting. This included health and social interventions, which were carried out without a pre-defined duration in time, mainly by nurses, community care officers and social workers. These interventions supported individuals in the activities of daily living. They were provided on the basis of an individual plan, implemented by the same multidisciplinary team, and managed by the same nurse "case manager" that had intervened in the intermediate phase.

In the case of routine care, patients were discharged from hospital to home after the needs assessment, and received the usual health and social care they would ordinarily receive. At home, they were periodically visited by their general practitioner, nurses, physiotherapists, geriatricians, community care officers and social workers. Patients received nursing interventions of varying levels of complexity and frequency, and the appropriate social care, without the coordination of a specific nurse "case manager". When required, medical specialists provided their services. In case of palliative care a nurse specialized in palliative treatments was added to the nursing team.

### Data sources

Data were extracted from different electronic databases that included:

- Hospital discharge records of S. G. Bosco Hospital containing International Classification of Diseases IX-Clinical Modification (ICD IX-CM) pathology codes and readmissions date;

- Data on discharge planning of the Operative Care Centre of Local Health Unit N°4 of Turin containing demographic variables (gender, age, etc) as well as physical and mental disability scales, such as ADL, IADL, and SPMSQ;

- Data from the registrar's office of the municipality of Turin in order to verify deaths and date of event;

- Data from the social services of the municipality of Turin containing social variables (family network, pension, etc).

Information on socio-demographic characteristics was collected for the following variables: gender, age at hospital admission (64–74, 75–84, 85+), living arrangements (living alone, living with at least a relative, caregiver), pension (< 750 euro, ≥ 750). Information on care needs, evaluated before hospital discharge, was categorised as follows: ADL scale (independent, partially dependent, and heavily dependent), IADL scale (independent, partially dependent, heavily dependent), and SPMSQ scale cognitive deterioration (absent-light, moderate and severe). The main reason for hospitalization was coded using the ICD-IX CM and then categorised according to the Major Disease Category in cardio-circulatory diseases, injury and poisonings, cancer, diseases of the respiratory system, and diseases of the digestive system. A further category was created for diseases not included in the previous Major Disease Category. The prescription and implementation of LTCI were derived from Operative Care Centre archives.

### Statistical analyses

Descriptive and inferential analyses were performed using SPSS 13.0. All subjects were followed up for a minimum of 6 months. Log-rank test with significance level of alpha = 0.05 was used to evaluate associations between type of care and each dependent variable (mortality and readmission) over the follow-up period. Two separate Cox regression analyses were applied to estimate adjusted Hazard Ratio (HR) with 95% Confidence Interval (95% CI) of death and hospital readmission, respectively. The variables that were significant at the univariate analysis at the alpha = 0.20 level were included in the Cox regression models. The p-value of log partial ratio test was evaluated to assess the significance of fitted models. Assumptions of hazards proportionality for Cox regression model were checked by Schoenfeld residuals and Log-Minus-Log plots.

Since the follow-up period was longer than the duration of stay in DPCH (i.e. 30 days at maximum), we took into account a relevant factor intervening after this period, which might have affected our outcome measures, that is the implementation of a LTCI plan. Therefore, the comparison is not limited to two groups (i.e. DPCH vs. routine care) but instead it is made among three groups (two subgroups of DPCH, according to the implementation of a long-term care plan during the follow-up period, vs. routine care).

### Ethics

Approval of the ethics committee was not required for the study. Data were extracted from routinely collected administrative databases and there was no need to obtain additional data from individual patients. The interventions under study were performed in ordinary or "natural" conditions, irrespective from the conduct of the present study. Because this was an observational retrospective study, patients had already been treated when the study protocol was written. Data linkage was performed by the team directly involved in patients' care using numerical codes. For the present study, researchers had access only to an anonymous dataset, which ensured patients' privacy. For these reasons, no personal informed consent to the present analysis was requested from study participants.

## Results

The socio-demographic characteristics of patients are presented in Table 1. This Table shows the number and percentages of subjects according to socio-demographic variables, reason for hospital admission and functional status, among the three study groups. In the overwhelming majority of subjects (94.4%) who received routine care no LTCI was implemented. The majority of subjects were elderly aged 75–84 years: 36.4% of those who received routine care, 42.4% in those admitted to care-home followed by implementation of long term care, and 53.4% among subjects admitted to care-home not followed by implementation of long-term care. There were statistically significant differences in the main reason for hospitalization among the three groups (p < 0.001); cancer was more frequent among those who received routine care than among both subgroups of DPCH (29.0% vs. 9.1% and 8.0%), while injuries were dominant in DPCH (6.5% vs. 19.2% and 25.3%).

**Table 1:** Number and percentage of typology of intermediate care and characteristics of the subjects

		**DPCH**		
	**Routine care**	**Followed by LTCI**	**Not followed by LTCI**	**p-value (Chi-square test)**
			
	n = 107	n = 99	n = 174	
***Gender***				
*Female*	59 (55.1%)	66 (66.7%)	108 (62.1%)	0.228
*Male*	48 (44.9%)	33 (33.3%)	66 (37.9%)	
***Age (years)***				
*64–74*	34 (31.8%)	20 (20.2%)	23 (13.2%)	0.003
*75–84*	39 (36.4%)	42 (42.4%)	93 (53.4%)	
*85+*	34 (31.8%)	37 (37.4%)	58 (33.3%)	
***Living arrangement***				
*Living alone*	44 (41.1%)	51 (51.5%)	91 (52.3%)	0.427
*Living with at least a relative*	48 (44.9%)	35 (35.4%)	63 (36.2%)	
*Caregiver*	15 (14.0%)	13 (13.1%)	20 (11.5%)	
***Pension***				
<*750 euro*	63 (58.9%)	51 (51.5%)	113 (65.3%)	0.08
≥ *750 euro*	44 (41.1%)	48 (48.5%)	60 (34.7%)	
***Primary diagnosis at admission***				
*Cardio-circulatory diseases*	26 (24.3%)	26 (26.3%)	46 (26.4%)	<0.001
*Injury and poisonings*	7 (6.5%)	19 (19.2%)	44 (25.3%)	
*Cancers*	31 (29.0%)	9 (9.1%)	14 (8.0%)	
*Diseases of the respiratory system*	9 (8.4%)	10 (10.1%)	21 (12.1%)	
*Diseases of the digestive system*	6 (5.6%)	7 (7.1%)	14 (8.0%)	
*Other diseases*	28 (26.2%)	28 (28.3%)	35 (20.1%)	
***ADL at hospital admission***				
*Independent*	10 (9.3%)	15 (15.3%)	12 (7.0%)	<0.001
*Partially dependent*	55 (51.4%)	79 (80.6%)	135 (78.9%)	
*Totally dependent*	42 (39.3%)	4 (4.1%)	24 (14.0%)	
***IADL at hospital admission***				
*Independent*	6 (5.6%)	13 (13.3%)	23 (13.4%)	0.001
*Partially dependent*	33 (30.8%)	50 (51.0%)	57 (33.1%)	
*Totally dependent*	68 (63.6%)	35 (35.7%)	92 (53.5%)	
***Cognitive deterioration***				
*Absent-light*	76 (71.0%)	79 (79.8%)	127 (73.4%)	0.165
*Moderate*	22 (20.6%)	18 (18.2%)	29 (16.8%)	
*Severe*	9 (8.4%)	2 (2.0%)	17 (9.8%)	

DPCH; discharge planning relying on a care-home model.

LTCI; long term care intervention.

ADL; activity of daily living.

IADL; instrumental activity of daily living.

As shown in Table 2, patients receiving routine care had higher crude mortality rates than those in routine care (45.8% vs. 10.1% and 22.4%, p < 0.001) after six months of follow-up. The difference was especially marked in the case of cancer (77.4% vs. 22.2% and 50.0%, p = 0.007) and cardio-circulatory diseases (42.3% vs. 7.7% and 13.0%, p = 0.002).

**Table 2:** Number and Percentage of deaths after six months of follow-up according to intermediate care typology and characteristics of subjects

		**DPCH**		
	**Routine care**	**Followed by LTCI**	**Not followed by LTCI**	p-value (Log-Rank test*)
			
	49 (45.8%)	10 (10.1%)	39 (22.4%)	
***Gender***				
*Female*	22 (37.3%)	4 (6.1%)	18 (16.7%)	0.002
*Male*	27 (56.3%)	6 (18.2%)	21 (31.8%)	
***Age (years)***				
*64–74*	18 (52.9%)	1 (5.0%)	3 (13.0%)	0.488
*75–84*	18 (46.2%)	4 (9.5%)	19 (20.4%)	
*85+*	13 (38.2%)	5 (13.5%)	17 (29.3%)	
***Living arrangement***				
*Living alone*	20 (45.5%)	5 (9.8%)	17 (18.7%)	0.570
*Living with at least a relative*	25 (52.1%)	3 (8.6%)	17 (27.0%)	
*Caregiver*	4 (26.7%)	2 (15.4%)	5 (25.0%)	
***Pension***				
<*750 euro*	28 (44.4%)	4 (7.8%)	26 (23.0%)	0.751
≥ *750 euro*	21 (47.7%)	6 (12.5%)	13 (21.7%)	
***Primary diagnosis at admission***				
*Cardio-circulatory diseases*	11 (42.3%)	2 (7.7%)	6 (13.0%)	<0.001
*Injury and poisonings*	0 (0.0%)	1 (5.3%)	8 (18.2%)	
*Cancers*	24 (77.4%)	2 (22.2%)	7 (50.0%)	
*Diseases of the respiratory system*	3 (33.3%)	1 (10.0%)	8 (38.1%)	
*Diseases of the digestive system*	2 (33.3%)	1 (14.3%)	2 (14.3%)	
*Other diseases*	9 (32.1%)	3 (10.7%)	8 (22.9%)	
***ADL at hospital admission***				
*Independent*	4 (40.0%)	1 (6.7%)	3 (25.0%)	0.030
*Partially dependent*	26 (47.3%)	7 (8.9%)	27 (20.0%)	
*Totally dependent*	19 (45.2%)	1 (25%)	9 (37.5%)	
***IADL at hospital admission***				
*Independent*	1 (16.7%)	1 (7.7%)	3 (13.0%)	0.324
*Partially dependent*	21 (63.6%)	3 (6.0%)	15 (26.3%)	
*Totally dependent*	27 (39.7%)	5 (14.3%)	21 (22.8%)	
***Cognitive deterioration***				
*Absent-light*	34 (44.7%)	8 (10.1%)	28 (22.0%)	0.603
*Moderate*	13 (59.1%)	2 (11.1%)	6 (20.7%)	
*Severe*	2 (22.2%)	0 (0.0%)	5 (29.4%)	

DPCH; discharge planning relying on a care-home model

LTCI; long term care intervention

ADL; activity of daily living

IADL; instrumental activity of daily living.

* Log-Rank test was applied within the whole follow-up period.

Table 3 shows the crude hospital readmission rates after six months of follow up for the three groups. About one in 5 subjects was re-admitted to the hospital within 6 months. Readmission was lower for subjects discharged to care-home (22.2% and 19.0% vs. 27.1%), but the difference was not statistically significant.

**Table 3:** Number and Percentage of Readmissions after six months of follow-up according to intermediate care typology and characteristics of subjects

		**DPCH**		
	**Routine care**	**Followed by LTCI**	**Not followed by LTCI**	p-value (Log-Rank test*)
			
	29 (27.1%)	22 (22.2%)	33 (19.0%)	
***Gender***				
*Female*	17 (28.8%)	11 (16.7%)	18 (16.7%)	0.129
*Male*	12 (25.0%)	11 (33.3%)	15 (22.7%)	
***Age (years)***				
*64–74*	8 (23.5%)	4 (20.0%)	2 (8.7%)	0.074
*75–84*	12 (30.8%)	12 (28.6%)	21 (22.6%)	
*85+*	9 (26.5%)	6 (16.2%)	10 (17.2%)	
***Living arrangement***				
*Living alone*	16 (36.4%)	10 (19.6%)	17 (18.7%)	0.393
*Living with at least a relative*	9 (18.8%)	9 (25.7%)	12 (19.0%)	
*Caregiver*	4 (26.7%)	3 (23.1%)	4 (20.0%)	
***Pension***				
<*750 euro*	14 (22.2%)	11 (21.6%)	23 (20.4%)	0.472
≥ *750 euro*	15 (34.1%)	11 (22.9%)	10 (16.7%)	
***Primary diagnosis at admission***				
*Cardio-circulatory diseases*	11 (42.3%)	4 (15.4%)	7 (15.2%)	0.256
*Injury and poisonings*	3 (42.9%)	4 (21.1%)	2 (4.5%)	
*Cancers*	6 (19.4%)	0 (0.0%)	4 (28.6%)	
*Diseases of the respiratory system*	3 (33.3%)	4 (40.0%)	7 (33.3%)	
*Diseases of the digestive system*	1 (16.7%)	3 (42.9%)	4 (28.6%)	
*Other diseases*	5 (17.9%)	7 (25.0%)	9 (25.7%)	
***ADL at hospital admission***				
*Independent*	3 (30.0%)	2 (13.3%)	2 (16.7%)	0.360
*Partially dependent*	17 (30.9%)	19 (24.1%)	27 (20.0%)	
*Totally dependent*	9 (21.4%)	1 (25.0%)	4 (16.7%)	
***IADL at hospital admission***				
*Independent*	2 (33.3%)	4 (30.8%)	2 (8.7%)	0.162
*Partially dependent*	8 (24.2%)	11 (22.0%)	17 (29.8%)	
*Totally dependent*	19 (27.9%)	6 (17.1%)	14 (15.2%)	
***Cognitive deterioration***				
*Absent-light*	20 (26.3)	15 (19.0%)	24 (18.9%)	0.103
*Moderate*	7 (31.8)	6 (33.3%)	4 (13.8%)	
*Severe*	2 (22.2)	1 (50.0%)	5 (29.4%)	

DPCH; discharge planning relying on a care-home model

LTCI; long term care intervention

ADL; activity of daily living

IADL; instrumental activity of daily living

*Log-Rank test was applied within the whole follow-up period.

The results of the multivariable Cox regression analyses are shown in tables 4 and 5. The independent predictors of mortality (Table 4) were cancer (HR = 3.27, 95% CI 1.93 – 5.55), diseases of respiratory system (HR = 1.84, 95% CI: 1.01 – 3.34), while DPCH followed by LTCI significantly decreased mortality compared to routine care (HR = 0.36; 95% CI 0.20–0.66). No significant difference was found between routine care and DPCH, when this was not followed by LTCI (HR = 1.15 95%CI: 0.77 – 1.74).

**Table 4:** Hazard Ratio of death

	**HR**	**95% CI**
***Gender***		
*Female*	1	
*Male*	1.44	1.00–2.09
***Age (years)***		
*64–74*	1	
*75–84*	0.77	0.48–1.26
*85+*	1.12	0.69–1.83
***Primary diagnosis at admission***		
*Cardio-circulatory diseases*	1	
*Injury and poisonings*	0.62	0.31–1.25
*Cancers*	3.27	1.93–5.55
*Diseases of the respiratory system*	1.84	1.01–3.34
*Diseases of the digestive system*	1.27	0.54–2.99
*Other diseases*	0.94	0.55–1.63
***ADL at hospital admission***		
*Independent*	1	
*Partially dependent*	0.57	0.26–1.24
*Totally dependent*	0.7	0.30–1.64
***IADL at hospital admission***		
*Independent*	1	
*Partially dependent and Totally dependent*	2.02	0.79–5.21
***Typology of term-care***		
*Routine care*	1	
*Care home not followed by long term care intervention*	1.15	0.77–1.74
*Care home followed by long term care intervention*	0.36	0.20–0.66
		
P-value of log partial likelihood ratio test <0.001		

HR; hazard ratio

CI; confidence interval

ADL; activity of daily living

IADL; instrumental activity of daily living.

**Table 5:** Hazard Ratio of hospital readmission

	**HR**	**95% CI**
***Gender***		
*Female*	1	
*Male*	1.43	0.94–2.16
***Age (years)***		
*64–74*	1	
*75–84*	1.4	0.81–2.40
*85+*	0.9	0.49–1.65
***Living arrangement***		
*Living alone*	1	
*Living with at least a relative or caregiver*	0.72	0.48–1.10
***Primary diagnosis at admission***		
*Cardio-circulatory diseases*	1	
*Injury and poisonings*	0.83	0.43–1.62
*Cancers*	0.99	0.49–2.00
*Diseases of the respiratory and digestive systems*	1.69	0.97–2.96
*Other diseases*	0.99	0.57–1.72
***IADL at hospital admission***		
*Independent*	1	
*Partially dependent*	1.47	0.70–3.07
*Totally dependent*	1.01	0.48–2.14
***Cognitive deterioration***		
*Absent-light*	1	
*Moderate*	1.31	0.78–2.17
*Severe*	2.2	1.09–4.43
***Typology of term-care***		
*Routine care*	1	
*Care home not followed by long term care intervention*	1.18	0.71–1.96
*Care home followed by long term care intervention*	1.01	0.48–2.14
		
P-value of log partial likelihood ratio test = 0.049		

HR; hazard ratio

CI; confidence interval

IADL; instrumental activity of daily living.

Table 5 shows HR and 95% CI for hospital readmission. Having a severe cognitive deterioration was a risk factor for readmission (HR = 2.20; 95% CI: 1.09–4.43). Both subgroups of DPCH showed similar hazards of readmission compared to routine care: HRs of hospital readmission were 1.01 (95%CI: 0.48 – 2.24) and 1.18 (95%CI: 0.71 – 1.96), for DPCH followed by LTCI and DPCH not followed by LTCI, respectively.

## Discussion

In our study, when socio-demographic factors, underlying disease and disability were taken into account, hospital discharge planning implemented in a residential care-home setting decreased mortality rates only if it was followed by a LTCI. On the other hand, adjusting for socio-demographic characteristics, health and functional status, readmission rates did not significantly differ among DPCH and routine care, irrespectively of the implementation of a LTCI.

About one in 5 patients (22.1%) was readmitted within 6 months from hospital discharge. This re-admission rate is aligned with that of Trappes-Lomax et al. [28]. To the best of our knowledge, no published study has so far assessed the effectiveness of DPCH taking into account the implementation of subsequent long-term care plans according to this logic of continuity of care. In this context, we chose an observational study design which is a very practical and useful research tool, given the complexity of the scenario. Consequently, our study may suffer from the typical limitations of observational studies, that is the incomparability of groups: since subjects were referred to the different types of care according to clinical judgements, social and organizational matters, systematic differences may have occurred in baseline characteristics of subjects. However, in the phase of data-analysis, we took into account some of the major confounders, that are socio-demographic, clinical and functional characteristics, by means of a multivariable regression model. The high death rate (77.4%) observed within 6 months from hospital discharge among cancer patients who received routine care may contribute to the different mortality experience of patients. However, similar results were found at stratified analyses. We also performed a multivariable regression analysis excluding cancer patients. The results confirmed the protective effect of DPCH when this was followed by a LTCI, with HR = 0.43 (95%CI: 0.22, 0.84).

Previous studies comparing residential care home intermediate services and routine care did not show differences on mortality and readmission rates after hospital discharge among elderly subjects. In one study, subjects who were referred to a care home rehabilitation service did not show reduced hospital readmission rates after 3 and 12 months of follow-up [33]. Similarly, no significant differences in the hazard of hospital readmission or death was found after 6 and 12 months of follow-up between a joint health/social care residential rehabilitation unit and "usual" care in the UK [28]. However, these studies do not report any information on any LTCI which the subjects may have received over the follow-up period. Lack of information on LTCI implemented over the follow-up and complementary with intermediate care services, does not permit a complete assessment of the appropriateness of the continuity of care. According to a recent review, continuity of care has two key elements: care of an individual patient and care delivered over time [34]. In particular, "management continuity" plays an important role especially in chronic or complex clinical diseases that require management from several providers. Shared management plans and care protocols facilitate management continuity, providing predictability and security in future care for both patients and providers. Therefore, in order to be maximally effective, "management continuity" should be planned systematically in advance, involving all the relevant actors in both interfaces of care. The first interface is the outward hospital interface, which is the transition from hospital to residential intermediate care-home services, while the second one refers to the transition from intermediate to long-term care.

Even when long-term care is deemed necessary, many different reasons may hinder its implementation, especially in the home setting. In our study, these were mainly related to financial difficulties, such as co-payment of social services, and organisational problems, such as delays in performing the multidimensional assessment, the existence of waiting lists for residential services and delays in the provision of home care. The availability of both intermediate care-home and long-term care services within the same facility, as shown in our study, might help overcome the aforementioned difficulties and create more confidence in patient and care givers. This is supported by the finding that proposed interventions of long-term care were more frequently provided if patients were referred to DPCH in comparison to routine care. In addition, this organisational formula may determine a better efficiency in the use of health care resources.

## Conclusion

The management of the continuity of care is fundamental especially in chronic or complex clinical diseases that require the contribution of several providers and personnel and are often implemented in different settings. Timely and shared plans ruling both the transitions between hospital and intermediate care, and between intermediate and long term care may determine better patients' outcomes. In this study, we attempted to open the "black box" of intermediate care, by describing context, setting and staffing involved. Future studies should focus on the evaluation of the effectiveness of hospital discharge planning taking into account the implementation of long-term care services.

## Abbreviations

DPCH: Discharge Planning relying on a Care-Home model; LTCI: Long Term Care Intervention; HR: Hazard Ratio; CI: Confidence Interval; ADL: Activity of Daily Living; IADL: Instrumental Activity of Daily Living; SPSMQ: Short Portable Mental Status Questionnaire

## Competing interests

The authors declare that they have no competing interests.

## Authors' contributions

All authors contributed to the conception of this paper, and to the acquisition of data. GD wrote the first draft and all authors made important contributions to subsequent drafts. All authors have seen and approved the final version. GD and BF had full access to all of the data in the study and take responsibility for the integrity of the data and the accuracy of the data analysis.

## Pre-publication history

The pre-publication history for this paper can be accessed here:


